# Ethnoveterinary Practices Related to Captive Elephants in Sauraha, Chitwan, Nepal

**DOI:** 10.1002/vms3.70435

**Published:** 2025-06-06

**Authors:** Sachin Devkota, Alok Dhakal, Sher Bahadur Jethara, Manish Chaudhary, Rakesh Kumar Yadav, Bijay Kumar Shrestha

**Affiliations:** ^1^ Paklihawa Campus Institute of Agriculture and Animal Science Tribhuvan University, Rupandehi Bhairahawa Nepal; ^2^ Department of National Parks and Wildlife Conservation Senior Veterinary Officer Chitwan National Park Ministry of Forests and Environment Government of Nepal Sauraha Nepal

**Keywords:** animal welfare, antimicrobials, captive elephants, ethnoveterinary medicine, livestock

## Abstract

The use of medicinal plants for treating animal diseases is a longstanding and widespread practice in Nepal, providing farmers with an accessible and cost‐effective option. This study aimed to document traditional knowledge regarding medicinal plants used to treat ailments in captive elephants in Sauraha, Chitwan. A total of 56 mahouts, responsible for the care of privately and government‐owned captive elephants, were interviewed through face‐to‐face interactions. The study identified 42 plant species from 26 families used to treat 27 ailments in captive elephants. Among these, the Fabaceae family was the most dominant, followed by the Poaceae family. The most commonly used plant parts were leaves, bark, and seeds. Medicinal herbs were predominantly prepared in paste formulations (21 plant species), while raw formulations were used for 15 species. The oral route of administration was the most popular method of application. The highest citation frequencies were recorded for *Azadirachta indica* A. Juss, *Brassica campestris* L., and *Trachyspermum ammi* (L.) Sprague. These findings highlight the high level of knowledge among mahouts regarding elephant ailments and their treatment using herbal plants. Given the risk of its loss, this traditional knowledgemust be throughly documented. These findings could provide meaningful insights for treating diseases in other animal species, contributing significantly to the field of ethnoveterinary medicine.

## Graphical abstract

The study documented 42 plant species from 26 families used for treating 27 ailments of captive elephants in Sauraha, Nepal.

Leaves were the most frequently used plant part, and the most common preparation method was paste formulation, followed by raw, juice, powder, roast, and decoction forms.

Oral route of administration was the most prevalent, followed by dermal and ocular routes.

Azadirachta indica (Neem) had the highest citation frequency, followed by Brassica campestris and Trachyspermum ammi.

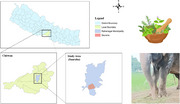

## 1 Introduction

Ayurveda is an ancient system of medicine for human health with historical roots in India (Mathpati et al. 2020). Ayurveda has been used to treat both human and animal diseases for centuries (Jaiswal and Williams 2017). The local plants utilised for treating diseases of livestock and domestic birds are generally called ethnoveterinary medicinal plants (Khattak et al. 2015). Traditional medical practices have been employed for generations and passed down orally from one generation to the next (Ouma 2022). Ancient books such as the *Agnipurana, Devipurana*, *Garudpurana, Lingapurana, Matsyapurana, Skandapurana, Charaka Samhita and Susruta Samhita* describe numerous methods of treating animal ailments using medicinal herbs (Upadhyay et al. 2011). However, these age‐old traditions are gradually fading as modern civilisation increasingly prioritises new technologies and medical advancements. The use of ethnoveterinary practice as primary care has been seen mostly in Asia and Africa, largely due to its cost‐effectiveness compared to pharmaceutical products. For example, in Nepal, researchers identified 103 plant species from 56 families used for treating domestic animals in the Argakhanchi district (Dhakal et al. 2021).

While ethnoveterinary practices have been well‐documented for various livestock species, there is a noticeable lack of information regarding their application to captive elephants. About 15,000 Asian elephants in range countries are utilised in temples, the timber industry, or kept under private ownership, while around 2,000 African and Asian elephants are housed in zoological facilities and circuses (Hildebrandt et al. 2012). Nepal is home to an estimated 400 elephants, with roughly equal numbers of wild and captive individuals found across the lowland regions (Ram and Acharya 2020). In such environments, health care and the dietary needs of captive elephants are often called into question. Studies on captive elephants in zoos, private facilities, and religious settings have highlighted concerns regarding their welfare and nutritional requirements (Vanitha et al. 2016; Dubost et al. 2019). So, as a cost‐effective alternative and complementary to modern medicines, most of the mahouts use ethnoveterinary practice for treatment of ailments. Few studies have documented the application of ethnoveterinary care in captive elephants. A study in India identified the use of 53 plant species from 29 families to treat 23 different diseases in captive elephants (Jayakumar et al. 2017).

To best of our knowledge, there has not been any documented ethnoveterinary study among mahouts in Nepal. This study aims to address this gap by documenting the use of ethnoveterinary medicine in the treatment of captive elephants in Sauraha, Chitwan, Nepal. This research not only aids in preserving the limited knowledge on elephant healthcare but also highlights the importance of preserving endangered plant species.

## 2 Methodology

### 2.1 Study Area

**FIGURE 1:**
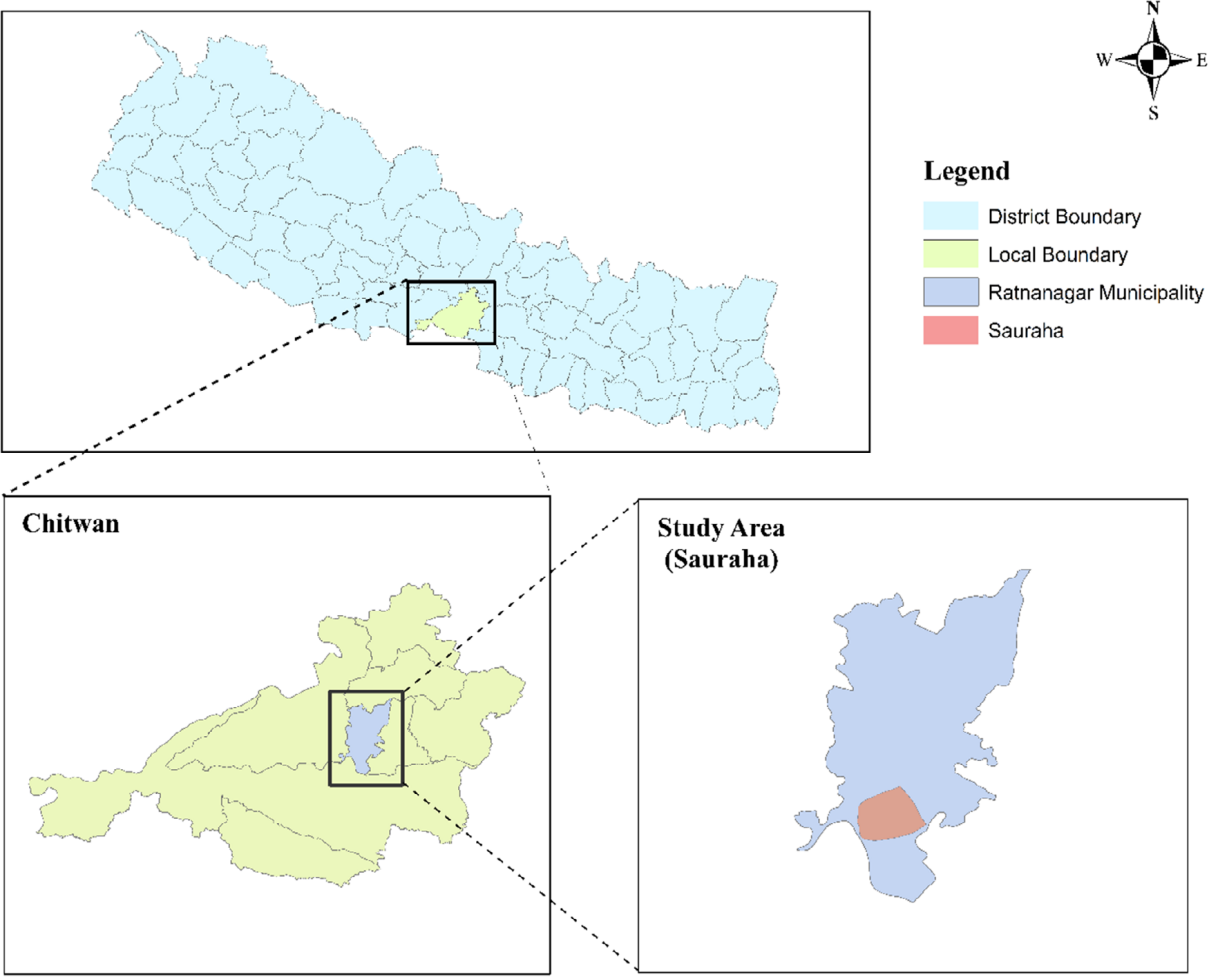
Map of study area showing Sauraha. The map was prepared using ArcGis 10.8, and the shape file was obtained from https://opendatanepal.com/dataset/new‐political‐and‐administrative‐boundaries‐shapefile‐of‐nepal.

The study was conducted in Sauraha, which lies in Ratnanagar municipality of Chitwan District, Nepal. The site was selected purposively, as Sauraha is the buffer zone of Chitwan National Park (CNP) (Figure 1). Sauraha is one of the major tourist destinations and has a high number of captive elephants. Sauraha is part of the subtropical Terai region and is characterised by diverse vegetation. The area is dominated by tropical moist deciduous forests, with Sal (*Shorea robusta* Roth) being the primary tree species. Riverine forests along the Rapti and Narayani rivers feature trees like Sissoo (*Dalbergia sissoo* Roxb.) and Khair (*Acacia catechu* (L.f.) Willd.). The region also includes grasslands, known as ‘Phanta’, which are rich in tall grasses such as *Saccharum spontaneum* (L.). This diverse vegetation supports a variety of wildlife, including elephants and one‐horned rhinoceroses, and is crucial to the local ecosystem and community livelihoods. CNP was established in 1973 and covers about 952.6 square km of land. Sauraha is located in the eastern part of CNP and is the park's key gateway. The CNP is situated on the southern portion of the Chitwan District sharing the southern frontier with Balmiki Tiger Reserve of India and the eastern boundary with Parsa National Park. The national park falls within the bio‐climatic sub‐tropical monsoonal climate, and the mean annual rainfall is 2,100 mm.

### 2.2 Field Work and Collection of Data

All mahouts taking care of private and government‐owned captive elephants in CNP were interviewed personally via face‐to‐face interview from July to September 2023. CNP and United Elephant Cooperative Limited provided information on details of captive elephants and mahouts. The interview was carried out using well‐prepared questionnaires consisting of both open‐and‐close ended questions. The questionnaires were prepared based on previous studies and knowledge of investigators (Dhakal et al. 2021). Participation in the study was voluntary, verbal informed consent was obtained from the participants, and they could withdraw from the survey anytime.

### 2.3 Plant Specimen Collection, Herbarium Preservation and Identification

Along with the survey with mahouts, the voucher specimens collected were transported to the lab for herbarium specimen preparation, a process which involved pressing the plants, drying them, and mounting them on appropriate herbarium sheets for permanent storage and future reference. The majority of plants were identified in the field, while some required further identification using available literature. Nomenclature and classification of the identified species were based on the following references (IUCN Nepal 2000; Aryal et al. 2016; POWO 2017; WFO 2023).

### 2.4 Data Analysis

Utilising MS Excel 2016, data were summarised for life form, plant parts used, application form, route of administration and citation frequency of plants. The formula used to calculate the citation frequency (Singh et al. 2012) was:

(i) $$\begin{array}{ccc} & & \mathrm{Citation}\;\mathrm{frequency}\;\left(C.f.\right)\\ & & \;=\;\frac{Number\;of\;times\;particular\;speices\;is\;mention}{Total\;number\;of\;informants}\;*100 \end{array}$$

## 3 Results

### 3.1 Socio‐demographic Profile of the Mahouts

Out of 56 mahouts, nearly all of them were male (98.2%), and there was only one female. Most (31/56) were below 40 years. Among the mahouts, 55.6% (30/56) were working for privately owned elephants, while 44.4% (26/56) were working for government‐owned captive elephants. About 50% (28/56) of them had work experience ranging from 10 to 20 years. Additionally, 66.1% (37/56) had experience of attending the elephant handling training programme (Table 1).

**TABLE 1:** Socio‐demographic profile of the mahouts.

Description	Percentage (Frequency)
**Age**	
<40	55.4%
≥40	44.6%
**Gender**	
Male	98.2%
Female	1.8%
**Years of experience**	
≤ 10	17.9%
11–19	50%
≥20	32.1%
**Attended elephant handling training program?**	
Yes	66.1%
No	33.9%
**Working for**	
Government owned captive elephants	55.6%
Private elephants	44.4%

### 3.2 Plant Diversity and Their Uses

A total of 42 plants were identified in the study area for treating different ailments of captive elephants (Table 2).

**TABLE 2:** List of plants used for ethnoveterinary medicine in study area.

ID	Family	Botanical name	Local name	Life form	Parts used	Form	Route	Ailment category	Ailments
C‐2023‐11	Acoraceae	*Acorus calamus* L.	Bojho or Ghodbachh	Herbs	Seed, leaf	Powder, paste	Dermal	Dermatological	Foot rot
					Leaf, root	Paste, roast	Oral	Gastro‐intestinal	Bloat^1^, indigestion, constipation^2,12^, stomach aches^3,12^, flatulence, diarrhea^11^
					Root	Roast	Oral	Respiratory	Cold
C‐2023‐50	Amaryllidaceae	*Allium cepa* L.	Pyaj	Herbs	Bulb	Paste	Oral	Gastro‐intestinal	Diarrhea^4^
								Ecto‐Endo parasite	Endo‐parasite^5^
C‐2023‐13		*Allium sativum* L.	Lasun	Herbs	Bulb	Paste, Roast	Oral	Gastro‐intestinal	Constipation^2,6,12^, stomach aches^12^, bloat^6^, diarrhea^7,11^
						Paste	Dermal	Wound and injury	Wound & injuries
							Oral	Reproductive	Musth^8^
							Dermal	Ecto‐endo parasite	Ecto‐parasite
C‐2023‐36	Apiaceae	*Elwendia persica* (Boiss.) Pimenov & Kljuykov	Kalo jeera	Herbs	Seed	Paste	Oral	Gastro‐intestinal	Stomach aches, indigestion, constipation^8^, flatulence^9^, bloat^9^
								Ecto‐endo parasite	Endo‐parasite
C‐2023‐12		*Cuminum cyminum* L.	Jeera	Herbs	Seed	Roast, paste, powder	Oral	Gastro‐intestinal	Constipation^10^, bloat, indigestion, stomach aches
C‐2023‐41		*Trachyspermum ammi* (L) sprague	Jwano	Herbs	Seed	Roast, paste	Oral	Gastro‐intestinal	Diarrhea^11^, constipation^10,12^, bloat, indigestion, stomach aches^12^, flatulence
						Juice	Oral	Respiratory	Common cold^13^
C‐2023‐22	Apocynaceae	*Calotropis gigantea* (L.) Dryand.	Aak	Shrubs	Root	Paste	Dermal	Dermatological	Foot Rot
					Flower	Roast	Oral	Respiratory	Common cold
C‐2023‐17	Asphodelaceae	*Aloe vera* (L.) Burm.f.	Musabbar or Ghyukumari	Herbs	Leaf	Decoction	Dermal	Inflammation	Swollen areas
						Paste	Dermal	Ecto‐Endo parasite	Ecto‐parasite
C‐2023‐23	Asteraceae	*Ageratum conyzoides* L.	Ganaune jhar	Herbs	Whole plant, Leaf	Juice, Paste	Dermal	Dermatological	Maggot infection, Foot Rot
C‐2023‐26	Brassicaceae	*Brassica campestris* L.	Tori	Herbs	Seed	Paste	Dermal	Dermatological	Foot Rot
							Dermal	Inflammation	Swollen^14^
							Dermal	Osteological	Swollen joint
							Dermal	Reproductive	Post‐partum massage
C‐2023‐40		*Rorippa indica* (L.) Hiern	Pahelo Jhar	Herbs	Whole plant	Paste	Oral	Ecto‐Endo parasite	Ecto‐parasite
C‐2023‐27	Convolvulaceae	*Cuscuta reflexa* Roxb.	Aakash Lati	Herbs	Whole plant	Juice	Dermal	Inflammation	Inflammatory parts
C‐2023‐43	Cucurbitaceae	*Benincasa hispida* Cogn.	Kuvindo	Climbers	Fruit	Raw	Oral	Reproductive	Musth
C‐2023‐31	Euphorbiaceae	*Trewia nudiflora* Wight.	Bhelar	Trees	Fruit	Paste	Dermal	Dermatological	Foot Rot
C‐2023‐24	Fabaceae	*Butea monosperma* (Lam.) Kuntze	Palas	Trees	Bark	Powder	Dermal	Dermatological	Maggot infection
C‐2023‐44		*Mimosa pudica* L.	Lajawati jhar	Herbs	Leaf	Raw	Oral	Gastro‐intestinal	Indigestion
C‐2023‐47		*Senegalia catechu* (L.f.) P.J.H.Hurter & Mabb.	Khair	Trees	Bark, Whole plant, Root	Raw, Powder	Oral	Gastro‐intestinal	Diarrhea, Indigestion
C‐2023‐14		*Tamarindus indica* L.	Imli	Trees	Leaf	Juice	Ocular	Eye problem	Abnormal eye discharges^15^
						Paste	Dermal	Osteological	Sprain
C‐2023‐25		*Trigonella foenum‐graecum* L.	Methi	Herbs	Seed	Decoction	Dermal	Inflammation	Swollen area^14,16,20^
						Powder	Oral	Gastro‐intestinal	Constipation^2,10,12^, Stomach aches^3,12^
C‐2023‐35	Gentianaceae	*Swertia chirayta* (Roxb.) H.Karst.	Chiraita	Herbs	Whole plant	Raw	Oral	Ecto‐Endo parasite	Endo‐parasite
C‐2023‐32	Lamiaceae	*Mentha spicata* L.	Pudina	Herbs	Leaf	Paste	Oral	Gastro‐intestinal	Constipation, Stomach aches, Indigestion
C‐2023‐33		*Ocimum tenuiflorum* L.	Tulsi	Shrubs	Leaf	Juice	Oral	Respiratory	Common cold
C‐2023‐16	Lauraceae	*Litsea glutinosa* (Lour.) C.B.Rob	Medh	Trees	Bark	Paste	Dermal	Osteological	Fracture
								Dermatological	Foot rot
C‐2023‐42	Malvaceae	*Bombax ceiba* L.	Simal	Trees	Bark	Raw	Oral	Respiratory	Cold
C‐2023‐19	Meliaceae	*Azadirachta indica* A.Juss.	Neem	Trees	Leaf, Bark	Paste	Dermal	Dermatological	Foot Rot
						Paste, Powder, Roast	Oral, Dermal	Ecto‐Endo parasite	Ecto‐parasite
						Raw	Oral	Gastro‐intestinal	Indigestion
						Juice	Dermal	Inflammation	Swollen areas^17^
						Juice	Dermal	Nervous	Paralysis of leg
						Powder, Paste	Dermal	Wound and injury	Wounded part
C‐2023‐49	Menispermaceae	*Tinospora cordifolia* (Willd.) Miers ex Hook.f. & Thomson	Gurjo	Climbers	Stem, Whole plant	Raw	Oral	Gastro‐intestinal	Diarrhea
						Raw	Oral	General weakness	Weakness
						Juice, Raw	Oral	Respiratory	Common cold^18^
C‐2023‐39	Moraceae	*Ficus benghalensis* L.	Bar	Trees	Bark, Leaf	Raw, Paste	Oral	Gastro‐intestinal	Diarrhea, Constipation, Bloat
						Raw	Oral	Respiratory	Cold
C‐2023‐37		*Ficus nemoralis* Wall. ex Miq.	Dudhe Lahara	Trees	Leaf, Bark	Paste, Roast	Dermal	Dermatological	Foot Rot
						Raw	Oral	Gastro‐intestinal	Bloat
C‐2023‐38		*Ficus religiosa* L.	Pipal	Trees	Leaves, Bark	Paste, Raw	Oral	Gastro‐intestinal	Diarrhea
							Oral	Respiratory	Cold
C‐2023‐21	Myrtaceae	*Psidium guajava* L.	Amba	Trees	Leaves	Raw	Oral	Gastro‐intestinal	Diarrhea
C‐2023‐52		*Syzygium cumini* (L.) Skeels	Jamun	Trees	Bark	Raw	Oral	Gastro‐intestinal	Diarrhea
C‐2023‐45	Piperaceae	*Piper nigrum* L.	Kalo Mirch	Shrubs	Seed	Paste	Oral	Gastro‐intestinal	Flatulence
						Powder	Oral	Reproductive	Musth^19^
C‐2023‐46	Poaceae	*Bambusa vulgaris* Schrad.ex.J.C.Wendl	Baas	Shrubs	Leaf	Raw	Oral	Gastro‐intestinal	Diarrhea
						Decoction	Dermal	Inflammation	Swollen areas^20^
						Raw	Oral	Reproductive	Normal parturition
						Decoction	Oral	Respiratory	Common cold^21^
C‐2023‐29		*Cynodon dactylon* (L.) Pers.	Dubo	Herbs	Leaf	Juice	Ocular	Eye problem	Redness of eye
C‐2023‐34		*Oryza sativa* L.	Dhan	Herbs	Seed	Raw	Oral	Gastro‐intestinal	Diarrhea
						Raw	Oral	Reproductive	Increase lactation^22^
C‐2023‐28		*Saccharum officinarum* L.	Ukhu	Shrubs	Fruit	Raw	Dermal	Dermatological	Maggot infection
					Stem	Raw	Oral	General weakness	Weakness
C‐2023‐48	Rutaceae	*Citrus limon (L.)* Burm. f.	Kagati	Shrubs	Fruit	Juice	Oral	Gastro‐intestinal	Constipation, Bloat
C‐2023‐51	Santalaceae	*Viscum album* L.	Harchul	Shrubs	Bark	Decoction	Dermal	Osteological	Fracture
C‐2023‐18	Solanaceae	*Physalis peruviana* L.	Khatmiti	Herbs	Leaf	Juice	Ocular	Eye problem	Cataract
C‐2023‐15	Vitaceae	*Cissus quadrangularis* L.	Mane	Climbers	Bark	Decoction	Dermal	Inflammation	Swollen parts
								Nervous	Paralysis, Nerve injury
								Osteological	Fracture, Swollen joint
						Powder		Wound and injury	Wounded region
C‐2023‐20	Zingiberaceae	*Curcuma longa* L.	Besar	Herbs	Rhizome	Paste, Decoction	Dermal	Dermatological	Foot Rot
							Oral	Gastro‐intestinal	Bloat^1^, diarrhea^7^, Constipation^12^, Stomach aches^12^
							Oral	Reproductive	Irregular estrous^23^
							Dermal	Wound and injury	Wounded parts
							Oral	Respiratory	Cold
C‐2023‐30		*Zingiber officinale* Roscoe	Adhuwa	Herbs	Rhizome	Powder	Oral	Gastro‐intestinal	Constipation^12^, Stomach aches^12^
						Roast		Respiratory	Cold^13^

1 = Used in combination of *Curcuma longa* and *Acorus calamus*, 2,3 = Used in combination with *Acorus calamus*, *Trigonella foenum‐graecum* and *Allium sativum*, 4, 5, 6 = mixed with yogurt, 7 = used in combination with *Curcuma longa* and *Allium sativum*, 8 = mixed with yogurt, 9 = Exercise of elephant done after administration, 10 = used in combination of *Cuminum cyminum*, *Trigonella foenum‐graecum* and *Trachyspermum ammi*, 11 = Used in combination with *Acorus calamus*, *Allium sativum* and *Trahyspermum ammi* along with Himalayan black salt (*Birenun*), 12 = Used in combination with *Trigonella foenum‐graecum*, *Allium sativum*, *Acorus calamus*, *Curcuma longa* and *Zingiber officinale*, 13 = used along with *Zingiber officinale* and *Trachyspermum ammi*, 14 = used along with *Brassica campestris* and *Trigonella foenum‐graecum*, 15 = leaves are soaked with salty water, 16 = seeds are fried in oil, 17 = boiled along with Camphor, 18 = used with honey, 19 = mixed with salt, 20 = used in combination with *Bambusa vulgaris* and *Trigonella foenum‐graecum* along with Camphor, 21 = feed with honey, 22 = used with Jaggery (*Bheli*), 23 = paste is mixed with Camphor and Himalayan black salt (*Birenun*).

### 3.3 Family of Plants and Their Life Form

The taxonomic diversity of plants that were reported are presented in Table 3. The present study indicates that most plant species used were herbs (20 species), followed by trees (12 species), shrubs (7 species), and climbers (3 species), respectively.

**TABLE 3:** Taxonomic diversity of plants.

Family	Number of plants
Fabaceae	5
Poaceae	4
Apiaceae	3
Moraceae	3
Amaryllidaceae	2
Brassicaceae	2
Lamiaceae	2
Myrtaceae	2
Zingiberaceae	2
Acoraceae	1
Apocynaceae	1
Asphodelaceae	1
Asteraceae	1
Convolvulaceae	1
Cucurbitaceae	1
Euphorbiaceae	1
Gentianaceae	1
Lauraceae	1
Malvaceae	1
Meliaceae	1
Menispermaceae	1
Piperaceae	1
Rutaceae	1
Santalaceae	1
Solanaceae	1
Vitaceae	1

### 3.4 Plant Parts Used of Medicinal Plants

The present study indicates that the most frequently used plant part was the leaf (of 15 plant species), followed by bark (of 11 plant species), seed (of 8 plant species), whole plant (of 6 plant species), fruit (of 4 plant species), root (of 3 plant species), bulb, rhizome, stem (of 2 plant species each) and flower (of 1 species).

### 3.5 Preparation Technique

In our study, 21 plant species were found to be applied as paste formulations, while 15 plant species were employed as raw formulations. These were followed by juice (10 species), powder (9 species), roast (8 species), and decoction formulation (6 species).

### 3.6 Route of Administration

The present study indicates that the most common route of administration was oral (28 species), followed by dermal (19 species) and ocular (3 species).

### 3.7 Citation Frequency (C.f.)

The highest citation frequency was found for *Azadirachta indica* (33.9), followed by *Brassica campestris* (32.1), and *Trachyspermum ammi* (25). The top 10 plant species with the highest citation frequency are provided in Table 4.

**TABLE 4:** Top 10 plants with maximum citation frequency.

Botanical name	Local name	Number of informants who cite the species	Citation frequency
*Azadirachta indica*	Neem	19	33.93
*Brassica campestris*	Tori	18	32.14
*Trachyspermum ammi*	Jwano	14	25.00
*Senegalia catechu*	Khair	13	23.21
*Bambusa vulgaris*	Baas	13	23.21
*Curcuma longa*	Besar	8	14.29
*Cissus quadrangularis*	Mane	7	12.50
*Tinospora cordifolia*	Gurjo	7	12.50
*Acorus calamus*	Bojho	7	12.50
*Allium sativum*	Lasun	6	10.71

## 4 Discussion

The mahouts are close to elephants and are responsible for their care and management. Proper training and experience are required for handling of captive elephants. During interviews, the mahouts shared that they received training from experienced animal handlers, animal welfare organisations, and veterinary technicians. These trainings often occurred during elephant health camps or other elephant‐focused programmes organised by Chitwan National Park.

The application of ethnoveterinary medicine is an important component in the treatment of ailments in captive elephants, which has also been discussed by Dubost et al. (2022). The herbal plants and their methods of preparation should be documented on a regular basis. In our study, a total of 42 plant species from 26 families were used in ethnoveterinary medicine that mahouts collected from forests and nearby places. In Thailand, mahouts integrated traditional healing methods such as herbal remedies, forest browsing, and spiritual practices into elephant care (Kittisirikul et al. 2024). Similarly in Laos, interviewed mahouts expressed optimism regarding the health benefits of certain plants, believing that their consumption leads to improvements in the elephant's well‐being (Dubost et al. 2019). Fabaceae was found to be the dominating family with 5 plant species, which is consistent with the findings of other authors studying ethnoveterinary practices for domestic animals (Tabuti et al. 2003; Ali‐Shtayeh et al. 2016). This might be due to the high diversity of Fabaceae, as they allow themselves to adopt a wide range of environmental conditions. In contrast to our study, the study on captive elephants by Jayakumar et al. (2017) found Apiaceae as a dominating family. This difference in observation might be due to differences in study area along with their vegetation. We cannot omit cultural and traditional disparity between the respondents. The finding of herbs as the primary life form of plants was similar to the study conducted by other researchers (Jayakumar et al. 2017; Dhakal et al. 2021). This might be due to the ability of herbs for rapid growth and a short life cycle with a wider range of environmental tolerance compared to trees and shrubs. In addition to this, herbs are also easily found everywhere and have ease of collection. Leaves were extensively used for the preparation of remedies, which is similar to the finding of other researchers (Dhakal et al. 2021). Leaves might be preferred because they are easy to collect, comparable to parts like flowers, bark, roots and fruits. They might also have extra compounds because photosynthesis and the production of metabolites occur there (Ghorbani 2005). In contrast with our findings, Jayakumar et al. (2017) reported maximum usage of seed. Maximum use of paste formulation was similar to the findings of Jayakumar et al. (2017). Pastes are mostly found as one of the popular methods in ethnoveterinary practices, as they are easy to prepare using mortar and pestle. In most of the cases, paste is generally prepared by adding water or yogurt. Some people also add milk or honey to boost viscosity and mask odour (Islam et al. 2014). The maximal application of the oral route was similar to the findings of Dhakal et al. (2021) and Giday et al. (2009). This study did not find any evidence of nasal administration.

Jayakumar et al. (2017) found *Ferula assa‐foetida* as the highest cited plant. This might be related to differences in the research area. According to Khwairakpam et al. (2018), various parts of *Acorus calamus*, such as the leaves and rhizomes, have traditionally been utilised in various medicinal formulations to treat a range of ailments, including arthritis, neuralgia, diarrhoea, dyspepsia, kidney and liver disorders, eczema, sinusitis, asthma, fevers, bronchitis, hair loss, and other health conditions. Additionally, Oyawoye et al. (2022) have mentioned various ethnomedicinal uses and properties of *Allium cepa* and *Allium sativum*. *Cuminum cyminum* has also been found effective against various digestive ailments in captive animals (Jayakumar et al. 2017). *Aloe vera* has shown efficacy in reducing swelling (Dhakal et al. 2021). These findings are consistent with our research, which also highlights the medicinal properties of these plants. *Ageratum conyzoides* has been widely recognised for its ethnomedicinal applications, including antimicrobial, anti‐ulcer, anti‐inflammatory, anticancer, antiprotozoal, antidiabetic, spasmolytic and analgesic properties (Yadav et al. 2019). *Swertia chirayita* is a well‐known medicinal herb utilised in traditional medicine to treat various conditions such as leprosy, malaria, and fever (Poonam and Singh 2009). According to El Menyiy et al. (2022), *Mentha spicata* has demonstrated potential antioxidant, antidiabetic, anti‐inflammatory, and anticancer properties. *P. nigrum* has primarily been used to treat gastrointestinal issues, such as indigestion, bloating, diarrhoea, flatulence, and stomach aches. Additional applications included the management of infertility, lack of appetite, respiratory conditions, and skin disorders related to coughing and colds (Takooree et al. 2019). According to Del Río et al. (2004), *Citrus limon* is mainly consists of flavonoids, which are involved in a variety of biological effects, such as antiviral, antioxidative, anti‐inflammatory, antiallergic, antiproliferative, antimutagenic, and anticarcinogenic properties contributing mainly to gastrointestinal properties. Numerous pharmacological activities, such as antioxidant, antineoplastic, antiviral, anti‐inflammatory, antibacterial, antifungal, antidiabetic, anticoagulant, antifertility, cardiovascular protective, hepatoprotective, and immunostimulant activity, have been demonstrated by curcumin, one of the curcuminoids found in the rhizome (Omosa 2017).

*Azadirachta indica* contains various bioactive compounds, including azadirachtin, nimbin, sodium nimbinate, tannin, flavonoids, limonoids, saponin and quercetin (Islas et al. 2020). *Brassica* seeds contain a diverse array of phytochemical compounds, including phenolics, polyphenols, flavonoids, carotenoids, and phenolic acids such as zeaxanthin, lutein, and β‐carotene (Favela‐González et al. 2020). It also comprises phytosterols, chlorophyll, alkaloids, and glucosinolates, including indoles and isothiocyanates (Favela‐González et al. 2020). Studies have also identified a range of phytochemical constituents in *Trachyspermum ammi*, including carbohydrates, glycosides, saponins, and phenolic compounds. Additionally, volatile oils such as thymol, γ‐terpinene, para‐cymene, and α‐ and β‐pinene have been reported. The presence of protein, fat, fibre, and essential minerals like calcium, phosphorus, iron, and nicotinic acid has also been documented (Bairwa et al. 2012). Phyto‐chemical compounds present in *Allium sativum* are derived from sulphur‐containing compounds such as allicin, alliin, diallyl sulphide, diallyl disulphide, diallyl trisulphide, ajeone, and S‐allyl‐cysteine (Shang et al. 2019). Similarly, the key bioactive ingredients found in *Allium cepa* include total phenolics, total flavonoids, and quercetin and its derivatives (Kumar et al. 2022). Berberine, palmatoside and palmatine are some of the active compounds found in *Tinospora cordifolia* (Jain et al. 2021). Various phytochemicals, including phenylpropanoids, sesquiterpenes, and monoterpenes, along with xanthone glycosides, flavones, lignins, steroids, inorganic compounds, mucilage, volatile oils, and saponins, have been extracted from different parts of *Acorus calamus* (Khwairakpam et al. 2018). *Cuminum cyminum* serves as a rich source of various bioactive compounds, including alkaloids, flavonoids, and terpenoids, among others (Singh et al. 2021). The seeds of *Trigonella foenum*‐*graecum* are a rich source of vital protective nutrients and bioactive compounds, including galactomannans, flavonoids, coumarins, saponins, alkaloids, and essential oils, which offer significant health benefits both individually and in combination with other bioactives (Alu'datt et al. 2024).Turmeric contains curcuminoids, which are its primary bioactive compounds responsible for its health benefits. Other compounds, such as essential oils, flavonoids, alkaloids, and polysaccharides, also contribute to the medicinal properties of turmeric (De Oliveira Filho et al. 2021). The overall phytochemical analysis of *Cissus quadrangularis* revealed the presence of flavonoids, triterpenoids, alkaloids, saponins, iridoids, stilbenes, vitamins, steroids, and glycosides (Bafna et al. 2021). Future efforts should focus on sustainable approaches to ensure the health and well‐being of captive elephants.

## 5 Conclusion

We found wide use of ethnoveterinary practice in captive elephants in Sauraha, Chitwan. The present study documented 42 plant species from 26 families that were used in ethnoveterinary medicine. Leaves were the most frequently used plant parts, and paste formulation was the most common preparation technique. There is a high need for further research into these herbal remedies. This would not only contribute to drug development but also support the conservation of these vital plant species and the preservation of indigenous knowledge present within mahouts.

## Author Contributions

**Sachin Devkota**: conceptualisation, methodology, validation, formal analysis, investigation, resources, data curation, writing ‐ original draft, writing ‐ review and editing, visualisation. **Alok Dhakal**: conceptualisation, methodology, validation, formal analysis, investigation, resources, data curation, writing ‐ original draft, writing ‐ review and editing, visualisation. **Sher Bahadur Jethara**: formal analysis, investigation, resources, data curation, writing ‐ original draft, writing ‐ review and editing. **Manish Chaudhary**: formal analysis, investigation, resources, data curation, writing ‐ original draft, writing ‐ review and editing. **Rakesh Kumar Yadav**: conceptualisation, methodology, validation, formal analysis, investigation, resources, writing ‐ original draft, writing ‐ review and editing, visualisation, project administration. **Bijay Kumar Shrestha**: conceptualisation, methodology, validation, resources, visualisation, project administration

## Ethics Statement

The study was approved by the Nepal Veterinary Council (Ref. No. Ethical 36/2080/81).

## Consent

Individuals’ consent was received prior to collecting data.

## Conflicts of Interest

The authors declare no conflicts of interest.

## Data Availability

The data used to support the findings of this study are available from the corresponding author upon reasonable request.
